# Prosthetic Rehabilitation of Ocular Defect resulting from Pediatric Retinoblastoma

**DOI:** 10.5005/jp-journals-10005-1267

**Published:** 2015-02-09

**Authors:** Suma Janya, Priyanka Gubrellay, Anupam Purwar, Shally Khanna

**Affiliations:** Professor, Department of Prosthodontics and Maxillofacial Prosthetics MS Ramaiah Dental College and Hospital, Bengaluru Karnataka, India; Assistant Professor, Department of Prosthodontics and Maxillofacial Prosthetics RR Dental College and Hospital, Udaipur, Rajasthan, India; Assistant Professor, Department of Prosthodontics, Purvanchal Institute of Dental Sciences, Gorakhpur, Uttar Pradesh, India; Assistant Professor, Department of Oral Pathology and Microbiology, Purvanchal Institute of Dental Sciences, Gorakhpur, Uttar Pradesh, India

**Keywords:** Ocular defect, Orbital enucleation, Retinoblastoma, Quality of life.

## Abstract

Ocular defects result from tumor, congenital anomaly and external injury not only lead to serious impairment of function and esthetics but also make the patient psychologically disabled. Prosthetic rehabilitation attempts to restore these disfgurements may improve esthetic, level of function, general psychologic improvement and quality of life. This clinical report details an attempt to rehabilitate a pediatric patient who has undergone orbital enucleation resulting from retinoblastoma with the aid of custom ocular prosthesis using commercially available prefabricated eye shell.

**How to cite this article:** Janya S, Gubrellay P, Purwar A, Khanna S. Prosthetic Rehabilitation of Ocular Defect resulting from Pediatric Retinoblastoma. Int J Clin Pediatr Dent 2014; 7(3):209-212.

## INTRODUCTION

Ocular malignancies have the potential for producing gross disfgurement and dysfunction, among them retino-blastoma is the most common and aggressive primary ocular malignancy of infancy and childhood.^1^ Pediatric patients, who undergo removal of an eye due to invasive and malignant nature of eye cancer, can be classified as: evisceration where the contents of the globe are removed leaving the sclera intact, enucleation where the entire eyeball is removed after severing the muscles and the optic nerve and exenteration where the entire contents of the orbit including the eyelids and the surrounding tissues are removed.^2^ Anatomically, these defects occur in the horizontal plane of the middle third of the face including two main categories: midline and lateral defects. Midline defects refer to the complete or partial involvement of the nose, and/or upper lip.^3^ A lateral defect may include complete or partial content of the cheek and-or orbit.^4^ The challenge of restoring such defect has always perplexed head and neck surgeon, plastic surgeon and maxillofacial prosthodontist.^3^

This clinical report describes the use of prefabricated eye shell to develop a customized ocular prosthesis for rehabilitation of postenucleation of right eye in a pediatric patient.

## CASE REPORT

An 8-year-old female patient referred to the Department of Maxillofacial Prosthodontics, MS Ramaiah Dental College and Hospital, Bengaluru, India, in August 2012, from Minto Eye Hospital, Bengaluru, for prosthetic rehabilitation of right ocular defect. History of present illness discovered that she was diagnosed with retinoblastoma, after which enucleation of right eye was conducted from same hospital in 2010. On extraoral examination, the margins and base of clinical defect were free from signs of infammation and completely healed. In this line of treatment plan, patient's guardians were explained about the fabrication of ocular prosthesis, and an informed, consent was obtained.

## CLINICAL PROCEDURES

**Fig. 1:**
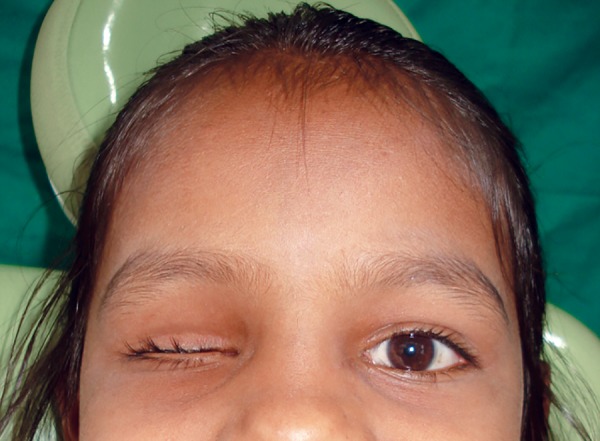
Preoperative view

**Fig. 2:**
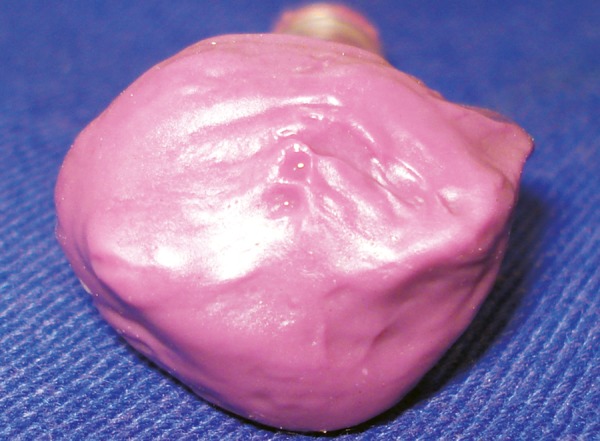
Final impression using polyvinyl siloxane impression material

**Fig. 3:**
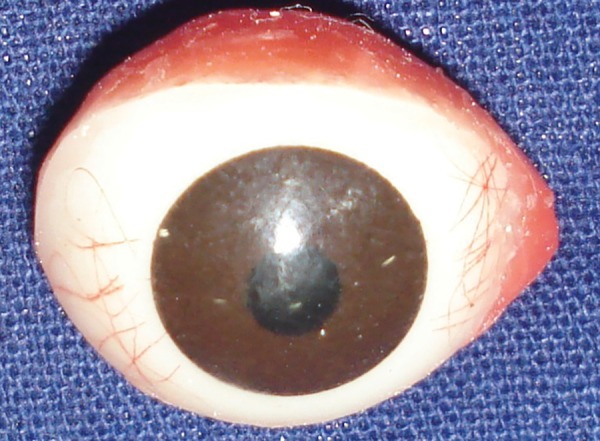
Wax pattern of sclera along with iris button

**Fig. 4:**
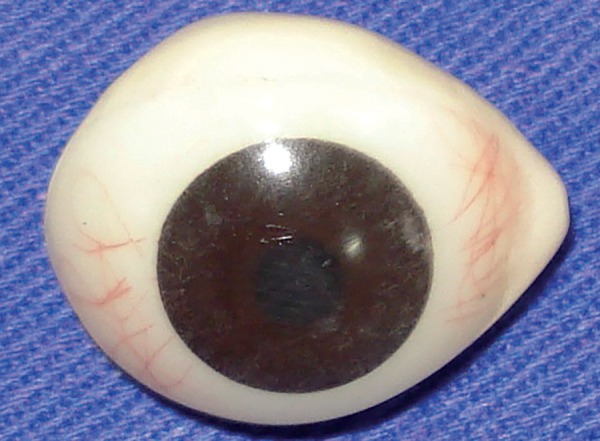
Definitive ocular prosthesis

**Fig. 5:**
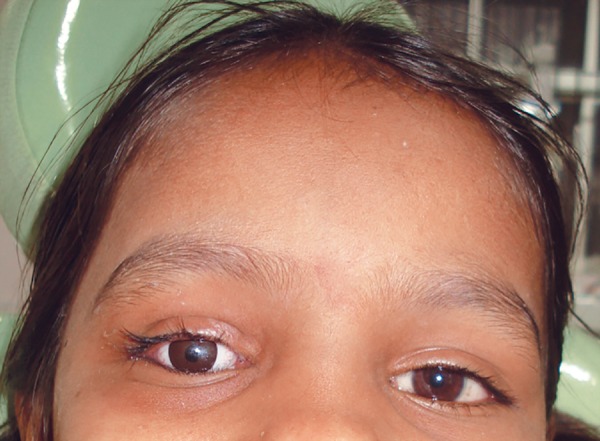
Postoperative view after immediate placement of ocular prosthesis

**Fig. 6:**
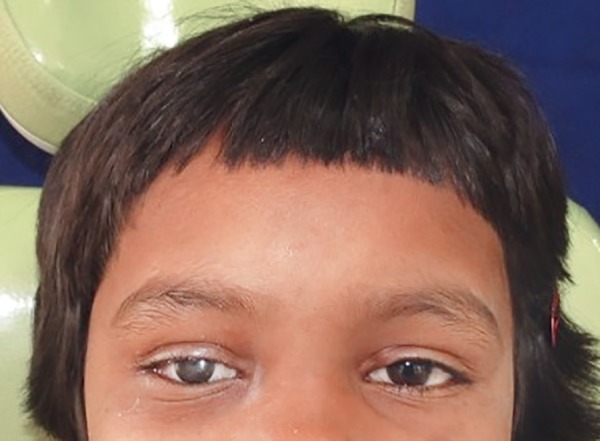
Postoperative view after 1 year follow-up

- The patient was seated in the physiological rest position, determined by the operator to make an impression of ocular defect (Fig. 1).
- The subject was draped with sterile hospital towel. Patient's eyebrow was lubricated the patient's eyebrow by light application of petroleum jelly for the easy removal of the impression material after it sets.
- Impression of affected area was made with the help of a disposable syringe cover and custom tray made of autopolymerizing resin tray (DPI-RR; Wallace Street, Mumbai, India). Extension of this resin tray was checked in patient's eye socket to prevent any irritation of soft tissue. Needle end cover was attached to resin tray and used for impression.
- This custom made resin tray was used as a suitable carrier for impression material (Reprosil, monophase; Dentsply International Inc, USA) to make fine and detailed functional impression of the tissue bed of eye socket. Automatic mixing gun system was used to inject the material into defect and simultaneously remaining impression material was loaded on to the impression surface of resin tray and passively placed in defect. Tray adhesive (Caulk, Dentsply International Inc, USA) was applied on the intaglio surface of resin tray for the retention of impression material.
- After the material was set, impression was removed gently (Fig. 2). Flasking of impression was performed by pouring lower half of impression in dental stone (Type III dental stone; Kalabhai Karson Ltd, Vikhroli (W), Mumbai, India). After dental stone was set, separating media (Cold mould seal, Dental Products of India Limited, Mumbai, India) was applied, and second mix of dental stone was poured. After fnal setting time of dental stone, both halves were separated and a wax pattern of sclera was fabricated with baseplate wax (The Hindustan Dental Products, Hyderabad, India). Wax pattern was added or trimmed from the basic sclera pattern until satisfactory contours of the eyelids were achieved in open and closed positions. A prefabricated eye shell or iris button selection done based on shape, size and color match with the contralateral eye was selected and positioned on the wax pattern (Fig. 3).
- The position of the iris was determined with the help of landmarks by making the patient look in a straight line. A vertical midline was marked passing through the forehead crease, glabella, tip of the nose and chin. The distance from the right eye medial canthus to the midline and left eye medial canthus to the midline was measured and calibrated.
- The fnished pattern was then fasked in a small two piece fask. Flasking was done taking care that the iris button should be secured to one counter of the fask and remaining part in the other portion of the fask. To achieve this acrylic stalk was attached to iris button before fasking. This acrylic stalk will ensure exact positioning of iris button. After dewaxing, packing was done with the selected heat-cure tooth color acrylic resin whose shade was matching with the sclera of contralateral eye (Fig. 4). Long cycle curing was done to avoid any residual monomer. The properly fnished and polished prosthesis was inserted in the socket after disinfection (Fig. 5).
- Patient and her guardians were instructed about hygiene practices (cleaning, placing and removal of the prosthesis). The need for regular recall appointments was emphasized to check the retention and comfort of prosthesis during observation period of 1 year (Fig. 6).

## DISCUSSION

Retinoblastoma is a most common, aggressive and invasive ocular malignancy of infancy and childhood. Survival and the chance of saving vision depend on severity of disease at presentation. Despite good understanding of its aetiology, mortality from retinoblastoma is about 70% in countries of low and middle income, where most affected children live.^1^

In the present case due to deep invasion of cancer, surgical excision of eye ball (enucleation) was conducted. Patient was kept under observation period for the complete healing of eye socket and referred to the department of maxillofacial prosthodontics for ocular rehabilitation. Occupational history of patient revealed that patient was a school-going child and hence it was an utmost social need of the patient to be esthetically acceptable by the society as early in life as possible. Hence, a custom ocular prosthesis using commercially available prefabricated eye shell was planned in order to enhance the confdence and quality of life of the patient.

Ocular prosthesis is broadly classified as stock,^5,6^ and custom made ocular prosthesis.^7-9^ Stock or prefabricated eye prosthesis has certain disadvantages of improper adaptation, compromised esthetic and limited eye movements where as custom made prosthesis advantages include improved adaptation to underlying tissues, increased mobility of the prosthesis, improved facial contours, and enhanced esthetics gained from control over the size of the iris and pupil, and color of the iris and sclera.^9^ It also involves technical sensitive procedures in various steps of fabrication which are quite difficult and based purely on artistic maneuverability of the maxillo-facial prosthodontist. Nevertheless, a custom prosthesis is more expensive than a stock prosthesis, and several steps are required for its fabrication. In our case, we have used prefabricated iris shell or iris button matched with patient contralateral eye and artificial custom made sclera was developed by heat-cure tooth color acrylic resin making it inexpensive and less time consuming. This combined approach for the development of ocular prosthesis surmounts the problem of prefabricated prosthesis and include the advantages of custom ocular prosthesis.

The scientific aspect requires an accurate impression for the development of an accurately fitting extraoral prosthesis.^10^ Various ocular impression techniques described so far have been based upon the materials available and the dexterity of the operator, making fabrication of an extraoral prosthesis more art than science.^11^ But each has its own integral advantages and disadvantages. Most can be placed into one of several broad categories: direct impression/external impression, impression with a stock ocular tray or modified stock ocular tray, impression using a stock ocular prosthesis, ocular prosthesis modif-cation and the wax scleral blank technique.^12^ In this case, impression with custom ocular tray was implemented. Miller suggested that a custom ocular tray is necessary in certain situations. For example, the anophthalmic socket could be highly irregular or stock trays may not be available.^13^

Today, numerous methods of retention for extraoral prostheses have been described in the literature; they include tissue undercuts, magnets and osseointegrated implants.^14^ Although osseointegrated implant may provide the most reliable prosthesis retention; additional surgeries, expenses, inadequate bone and prior radiation to the area may contraindicate this type of treatment.^15^ Inpresent case, retention was primarily achieved through anatomic tissue undercut.

A subjective measurement of patient was evaluated in this case to assess the improvement in quality of life (QoL). Chang et al proposed a standard questionnaire to evaluate the patient's satisfaction with facial prosthesis.^16^ Concept of QoL has emerged as an organizing scheme around which to describe and evaluate the experience of cancer patient in clinical research. In the described case, after one year of follow-up patient explained that retention and overall appearance was fair and satisfactory, wearing and cleaning of prosthesis was comfortable with no tissue discomfort was reported. This inferred that patient had overall improvement in QoL during observation period.

## CONCLUSION

Multidisciplinary approach is required to treat maxillo-facial rehabilitation cases. The use of custom-made ocular prosthesis has provided better option to the vast majority where implant is not a firsthand choice in developing countries due to its economic factor and other associated contraindications. In the described case, rehabilitation of pediatric patient is challenging as it require long-term follow-up till development of the enucleated socket is complete at the age of 12 years. In this long-observation period, childhood patient needs psychological support to recover confdence and self esteem in today's cosmetically challenging world.
